# Sarcoma of Jaw-Death

**Published:** 1880-04

**Authors:** 


### SARCOMA OF JAW—DEATH.

Dr. Erskine Mason presented a specimen showing sarcoma of the superior maxilla. A woman, aged twenty-four years, had an abscess of the face of short duration, which healed two years before she came under observation. After the abscess closed she noticed a node near the cicatrix, which increased in size until she was seen by Dr. Mason. The maxilla was found to be involved in a tumor, which was suspected to be a sarcoma. The eyeball on the affected side was slightly protruded. The most urgent symptom was distressing pain, and to relieve that the whole of the maxilla was extirpated. The wound readily healed. There was relief of pain and improvement of vision of the affected eye.

This continued for several months, the patient being apparently well. The tumor returned, however, and when examined it was found to have projected into the mouth. The most important symptom on the return, was epistaxis. Extirpation was again practiced, with benefit, the wound healing very kindly. Exhausting hemorrhages occurred with the second return of the tumor, which resulted in death. The autopsy showed that the growth was of the spindle-celled form of sarcoma. It penetrated into the nasal fossa, the pharynx, the orbit, and through the sphenoid bone into the middle of the skull. At no time were there any cerebral symptoms which might not readily be attributed to the exhaustion.

